# Impact of groundwater depth and soil salinity on riparian plant diversity and distribution in an arid area of China

**DOI:** 10.1038/s41598-020-64045-w

**Published:** 2020-04-29

**Authors:** Yong Zeng, Chengyi Zhao, Fengzhi Shi, Michael Schneider, Guanghui Lv, Yan Li

**Affiliations:** 1 State Key Laboratory of Desert and Oasis Ecology, Xinjiang Institute of Ecology and Geography, Chinese Academy of Sciences, Urumqi, 830011 Xinjiang China; 2 College of Resources and Environmental Sciences, Xinjiang University, Urumqi, 830046 Xinjiang China; 3 University of Chinese Academy of Sciences, Beijing, 100049 China; 4 Nanjing University of Information Science and Technology, Nanjing, 210044 China; 5 Earth Sciences, Freie Universität Berlin (FUB), Malteserstr. 74-100, 12249 Berlin, Germany

**Keywords:** Restoration ecology, Riparian ecology

## Abstract

Riparian plant diversity in arid regions is sensitive to changes in groundwater. Although it is well known that groundwater has a significant influence on plant diversity, there have been few studies on how groundwater and soil salinity impact plant community in desert riparian ecosystems. Therefore, we surveyed 77 quadrats (100 m × 100 m) to examine the relationship between groundwater depth, groundwater salinity, soil salinity and plant community in the upper reaches of the Tarim River. Data were analyzed with two-way indicator species analysis (TWINSPAN), detrended canonical correspondence analysis (DCCA) and principal component analysis (PCA). The results indicated that *Populus euphratica*, *Tamarix ramosissima*, and *Phragmites australis* were the dominant plants among trees, shrubs and herbs, respectively. Five plant community types were classified. There were significant differences in species diversity, soil moisture, soil salinity, groundwater depth and groundwater salinity across the community types. The composition and distribution of plant community are significantly influenced by groundwater depth, groundwater salinity, soil moisture, distances from the river to the quadrats, soil pH, electrical conductivity, total salt, CO_3_^2−^, Cl^−^, SO_4_^2−^, Ca^2+^, Mg^2+^, Na^+^ and K^+^. Shallow groundwater depth, low groundwater salinity, and high soil moisture and soil salinity were associated with higher plant diversity.

## Introduction

Riparian areas are in the transition zone between aquatic and terrestrial ecosystems and play a significant role in the energy and nutrient fluxes between the two types of ecosystems^1^. Riparian habitats comprise a diverse collection of valuable species and are regarded as biodiversity corridors^2^. Riparian vegetation plays an important role in protecting biodiversity, providing animal food and habitats for animals, and maintaining ecological balance^3^. However, riparian vegetation has become less stable as groundwater tables have dropped, leading to declines in arid desert river systems^4^. Therefore, the analysis of the changes in species composition and community distribution is crucial for protecting the biodiversity of riparian ecosystems^5^.

Riparian vegetation in arid regions is mainly controlled by precipitation, surface runoff, and groundwater^6^. High rates of evapotranspiration and low annual precipitation are characteristic of arid desert river basins^6^. The low precipitation and limited surface runoff, both spatially and temporally, in extremely arid regions do not provide any significant source of water for plant growth^7^. Thus, groundwater constitutes the main water source for vegetation in arid river ecosystems^8^.

Riparian plant species, as groundwater-dependent vegetation, are referred to as phreatophytes^9^. Riparian vegetation productivity, biomass, competitiveness, composition, structure, and abundance are controlled by the groundwater^10^. Increases in water table depth has imposed drought stress on vegetation and reduced plant cover, diversity and richness^11,12^. Salt accumulation associated with high rate of evaporation of shallow groundwater through the unsaturated zone has been shown to influence plant composition in many arid riparian systems^13^. The area proximity to river had high salt accumulation^12^. The soil moisture content, electrical conductivity and pH in the areas nearer to the riverbank were generally higher than other areas^14^. Riparian plant species richness was positively associated with high soil pH in a riparian forest^14,15^. Therefore, it is necessary to understand the relationship between groundwater, soil salinity and the plant community in arid riparian ecosystems.

The Tarim River, located in the Tarim Basin, which is the most arid basin in China, is 1321 km long and is the second largest sandy desert on earth^6,12^. For the period from 1957 to 2000, the average annual inflow along the upper and lower reaches was 4.74 km^3^/a and 1.42 km^3^/a, respectively, while the environmental flow was 1.65 km^3^/a and 0.18 km^3^/a^16^, respectively. However, due to the severe misuse of water resources, the annual run-off in the upper reaches of the Tarim River has declined^17^, causing a reduction in the groundwater level in the upper and lower reaches^18^. The forests along the lower reaches have already been strongly degraded or even completely destroyed^19^. To restore and reconstruct the natural degraded arid riparian ecosystems, a 1.8 billion US dollar water diversion project has been invested in by the Chinese government since 2000. The restoration effort has been successful within 800 m from the river channel^20^. The groundwater depth declined from 12.6 m to 5.5–6.2 m between 2000 and 2015 in the lower reaches^21^. The riparian ecosystem plays a significant role instabilizing the water balance of the desert oasis and limiting desertification^22,23^. Many studies have examined the relationship between community and groundwater depth in the lower reaches of the Tarim River^6,11,18^. For example, Hao *et al*.^6^ found that richness and diversity declined with increasing groundwater depth. Li *et al*.^24^ found that the community structure changed from trees/shrubs/herbs to trees/shrubs when the groundwater depth increased from shallow to deep. Although it is well known that groundwater has a significant influence on plant diversity, there have been few studies on how groundwater and soil salinity impact the plant community in desert riparian ecosystems. Furthermore, the upper reaches, constituting the core area of the Tarim River riparian zone, are less well studied^25^.

The objectives of the present study are (1) to characterize the plant composition and community along the upper reaches of the Tarim River and (2) to determine the influences of groundwater depth and soil salinity on the plant communities. Our study provides a scientific foundation for informing government decisions related to ecological protection in arid riparian regions.

## Results

### Plant community composition

The plant composition categories in the upper reaches of the Tarim River included trees, shrubs, and herbs (Table 1). Twenty-two species were found in the 77 investigated quadrats: 2 tree species, 7 shrub species, and 13 herbaceous species. In the tree layer, the relative density, relative frequency, and relative dominance of *P. euphratica* were larger than those of *P. pruinosa*, and the importance value of *P. euphratica* was 80.31%. In the shrub and herbaceous layers, *T. ramosissima* and *P. australis* possessed the largest importance values (65.71% and 26.67%, respectively).

**Table 1:** The important value index (IVI%), relative density (RD%), relative frequency (RF%), relative dominance/relative basal coverage (RDM%/RBC%) was calculated for each species at each tree, shrub and herb layer in 77 quadrats. IVI_Tree_ =(RD + RF + RDM)/3, IVI_Shrub or herb_ =(RD + RF + RBC)/3.

No.	Species	RD%	RF%	RDM/RBC%	IVI%	Rank
Tree layer		100	100	100	100	
1	*Populus euphratica*	76.71	80.00	83.29	80.31	1
2	*P. pruinosa*	23.22	19.18	16.66	19.69	2
Shrub layer		100	100	100	100	
1	*Tamarix ramosissima*	56.09	59.40	31.72	65.71	1
2	*T. hispida*	3.03	9.25	4.68	5.64	5
3	*T. arceuthoides*	10.64	1.79	8.81	7.07	4
4	*Haloxylon ammodendron*	0.08	2.09	0.14	0.76	7
5	*Lycium ruthenicum*	14.41	12.54	0.80	9.24	3
6	*Halimodendron halodendron*	0.15	2.99	0.06	1.05	6
7	*Halostachys caspica*	15.618	11.94	3.79	10.53	2
Herblayer		100	100	100	100	
1	*Glycyrrhiza inflata*	15.73	5.39	0.62	7.27	5
2	*Poacynum hendersonii*	1.27	2.70	0.87	1.50	9
3	*Hexinia polydichotoma*	0.08	1.08	0.02	0.40	11
4	*Karelinia caspia*	19.83	15.10	21.28	18.74	3
5	*Cynanchum sibiricum*	0.04	1.08	0.01	0.38	13
6	*Calamagrostis pseudophragmites*	1.80	1.62	1.15	1.53	10
7	*Inula salsoloides*	0.08	1.08	0.01	0.40	12
8	*Phragmites australis*	33.14	12.40	34.44	26.67	1
9	*Apocynum venetum*	5.63	6.47	20.16	10.76	4
10	*Alhagi sparsifolia*	17.42	31.27	20.95	23.22	2
11	*Halocnemum strobilaceum*	0.36	4.85	0.11	1.78	8
12	*Salsola ruthenica*	2.57	10.78	0.31	4.56	6
13	*Halogeton arachnoideus*	2.06	6.20	0.08	2.79	7

### Classification of the plant communities

**Figure 1:**
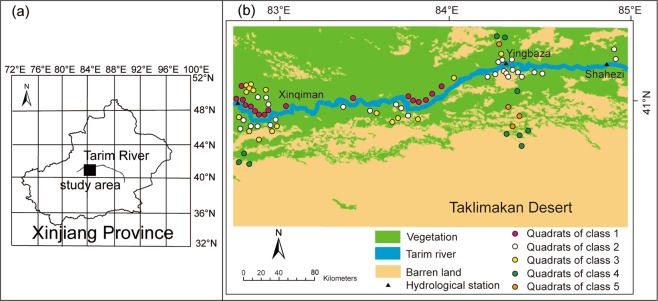
(**a**) location of the study area, and (**b**) spatial distribution of quadrats for five pant community classes as determined using the TWINSPAN clustering classifier in upper reaches of Tarim River. The source of map was from the resource and environment data cloud platform. The URL for the source of the map is http://www.resdc.cn/data.aspx?DATAID=184.

**Figure 2:**
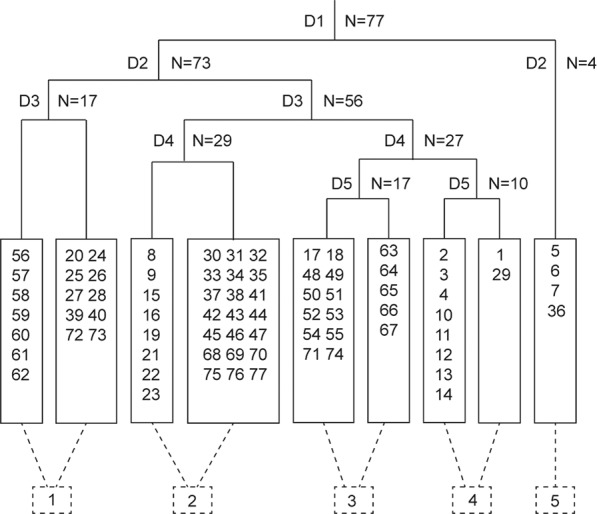
Dendrogram of TWINSPAN analysis classification of 77 quadrats in upper reaches of Tarim River. Arabic numbers in solid rectangle represent individual quadrats and arabic numbers in the dashed rectangle represent plant community classifications.

Five plant community classes were identified using TWINSPAN (Figs. 1b and 2; Fig. A1). Class 1: *P. euphratica* + *Tamarix* spp*., L. ruthenicum*, *H. caspica*, *H. halodendron* + *A. sparsifolia*, *K. caspia*, *H. strobilaceum*, *C. sibiricum*, *P. hendersonii*, *I. salsoloides*, and *H. polydichotoma*. *P. euphratica* (tree layer), *Tamarix* spp., *L. ruthenicum*, *H. caspica*, *H. halodendron* (shrub layer) and the herbaceous layer constitute the plant community (Table 2). Class 1 includes eight subclasses, with 17 plant quadrats that were mainly distributed in the direction of the oasis, close to the river channel (Fig. 1b).

**Table 2:** Five plant community class identified using TWINSPAN, community composition and number of quadrats.

Community classification	Sub-classes	Name	Number of quadrats
Class 1	1	*Pop eup* + *Tam his, Tam ram, Lyc rut, Hal cas* + *Alh spa, Karcas, Hal str*	1
	2	*Pop eup* + *Tam ram, Lyc rut, Hal cas* + *Alh spa, Karcas, Cyn sib*	2
	3	*Pop eup* + *Tam ram, Lyc rut* + *Alh spa, Karcas*, *Hex pol*	2
	4	*Pop eup* + *Tam ram, Lyc rut, Hal hal* + *Alh spa, Karcas, Hex pol, Poa hen, Apo ven, Inusal*	2
	5	*Pop eup* + *Tam ram, Lyc rut* + *Alh spa, Karcas, Hex pol*	1
	6	*Pop eup* + *Tam ram, Hal cas* + *Alh spa, Karcas*	2
	7	*Pop eup* + *Tam ram, Hal cas* + *Hal str, Alh spa*	3
	8	*Pop eup* + *Tam ram, Hal cas* + *Hal str*	4
Class 2	1	*Pop eup* + *Tam his, Tam ram* + *Alh spa, Hex pol*,	4
	2	*Pop eup* + *Tamarc* + *Alh spa, Karcas*,	2
	3	*Pop eup, Pop pru* + *Tam ram* + *Alh spa, Karcas,Poa hen, Cal pse, Inusal, Apo ven*	2
	4	*Pop eup, Pop pru* + *Tam ram* + *Alh spa, Karcas, Phraus, Poa hen, Cal pse, Apo ven*	2
	5	*Pop eup, Pop pru* + *Tam ram* + *Karcas, Phraus, Cal pse, Alh spa*	11
	6	*Pop eup* + *Tam his* + *Alh spa, Karcas*	4
	7	*Pop eup, Pop pru* + *Tam ram* + *Phraus*	4
Class 3	1	*Pop eup*, *Pop pru* + *Tam ram* + *Alh spa*	1
	2	*Pop eup*, *Pop pru* + *Tam ram* + *Glyinf*	4
	3	*Pop eup*, *Pop pru* + *Tam ram* + *Glyinf, Apo ven*	1
	4	*Pop eup,Poppru* + *Tam ram* + *Apo ven*	1
	5	*Pop eup* + *Tam ram* + *Glyinf*	4
	6	*Pop eup* + *Tam ram* + *Alh spa*	1
	7	*Pop eup* + *Tam ram* + *Alh spa*, *Apo ven*	1
	8	*Pop eup*, *Pop pru* + *Alh spa, Glyinf*	1
	9	*Pop eup*, *Pop pru* + *Apo ven*	1
	10	*Pop eup* + *Glyinf*	2
Class 4	1	*Tam ram*, *Hal amm* + *Hal ara*,*Sal rut*	3
	2	*Tam ram* + *Hal ara*, *Sal rut*	4
	3	*Tam ram* + *Sal rut*	1
	4	*Tam his* + *Hal ara*, *Sal rut*	1
	5	*Tam his*	1
Class 5	1	*Pop eup* + *Hal ara*, *Sal rut*	1
	2	*Pop eup* + *Sal rut*	1
	3	*Pop eup* + *Hal ara*	1
	4	*Sal rut*	1

Species names are abbreviated using the first three letters of genus and species names; full species names are listed in Table 1.

Class 2**:** *Populus* spp.+ *Tamarix* spp.+ *A. sparsifolia, H. polydichotoma, K. caspia, C. pseudophragmites, P. hendersonii*, and *P. australis. P. euphratica* and *P. pruinosa* (tree layer), *Tamarix* spp. (shrub layer) and the herbaceous layer constitute the plant community (Table 2). Class 2 includes seven subclasses, with 29 plant quadrats that were mainly distributed in the direction of the desert, close to the river channel (Fig. 1b).

Class 3: *Populus* spp. + *T. ramosissima* + *A. sparsifolia*, *G. inflata*, and *A. venetum*. *Populus*spp. (tree layer), *T. ramosissima* (shrub layer), and few herbaceous plants constitute the plant community (Table 2). Class 3 includes 10 subclasses, with 17 plant quadrats that were mainly distributed an average distance of approximately 5 km away from the river channel (Fig. 1b).

Class 4: *Tamarix* spp., *H. ammodendron* + *H. arachnoideus*, and *S. ruthenica*. This plant community comprises *Tamarix* spp., *H. ammodendron* (shrub layer) and few herbaceous plants (Table 2). Class 4 includes six subclasses, with 10 plant quadrats that were mainly distributed an average distance of approximately 23 km away from the river channel (Fig. 1b).

Class 5: *P. euphratica* + *H. arachnoideus*, and *S. ruthenica*. This plant community comprises *P. euphratica* (tree layer) and few herbaceous plants (Table 2). Class 5 includes four subclasses, with four plant quadrats that were mainly distributed an average distance of approximately 22 km away from the river channel (Fig. 1b).

### Plant diversity and environmental factors under different plant community types

**Figure 3:**
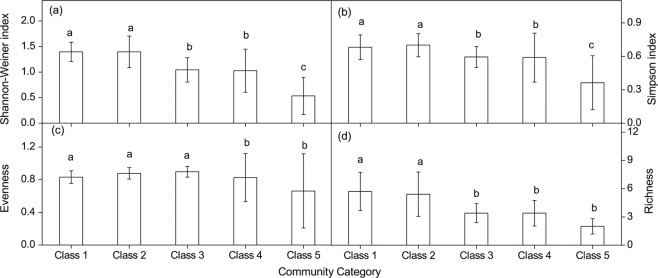
Plant diversity index of different community category (a Shannon-Wiener index, b Simpson index, c Species evenness, d Species richness).

Significant differences in the Shannon-Weiner index, Simpson index, evenness index, richness index, groundwater depth, distance from the river channel, soil pH, electrical conductivity, total salt, CO_3_^2−^, Cl^−^, and SO_4_^2−^ were found among the five plant community types (Fig. 3; Table 3). The values of the plant diversity indices, groundwater and soil salinity for each community were ranked from the highest to the lowest values. The plant diversity indices, soil moisture, pH, EC, TS, CO_3_^2−^, Cl^−^, and SO_4_^2−^ were ranked as follows: class 1, class 2, class 3, class 4, and class 5; distance from the river channel and groundwater depth: class 4, class 5, class 3, class 2, and class 1; groundwater salinity: class 5, class 4, class 3, class 2, and class 1.

**Table 3:** Groundwater and surface soil salinity of different community category.

Environmental parameters	Class 1	Class 2	Class 3	Class 4	Class 5
DistR. km	2.01 ± 1.77b	2.06 ± 1.69b	4.96 ± 1.76b	23.56 ± 8.65a	22.69 ± 5.70a
GWD. m	3.57 ± 0.68b	4.31 ± 1.46b	4.69 ± 1.49b	14.64 ± 5.63a	13.75 ± 6.24a
GS (g/L)	1.34 ± 0.19c	1.51 ± 0.26c	1.57 ± 0.91c	3.55 ± 1.24b	5.20 ± 0.75a
GEC (g/L)	1.42 ± 0.21c	1.51 ± 0.19c	1.74 ± 0.92c	3.63 ± 1.29b	5.52 ± 0.76a
SM (%)	19.67 ± 2.17a	11.10 ± 2.73ab	10.59 ± 2.10ab	3.61 ± 1.51b	2.79 ± 1.14b
TS (g/kg)	36.08 ± 8.34a	31.85 ± 9.30a	18.72 ± 2.93b	13.20 ± 2.56b	10.62 ± 1.28b
PH	8.76 ± 0.31a	8.69 ± 0.26a	8.26 ± 0.29b	8.14 ± 0.29c	7.80 ± 0.04c
EC (ms/cm)	10.26 ± 3.24a	8.91 ± 1.66a	3.88 ± 1.07b	3.31 ± 1.49b	2.61 ± 0.49b
CO_3_^2−^ (g/kg)	0.02 ± 0.004a	0.02 ± 0.013a	0.00 ± 0.001b	0.00 ± 0.001b	0.00 ± 0.000b
HCO_3_^−^ (g/kg)	0.20 ± 0.056a	0.21 ± 0.074a	0.22 ± 0.049a	0.20 ± 0.050a	0.18 ± 0.013a
Cl^−^ (g/kg)	15.37 ± 4.14a	12.64 ± 4.51a	8.43 ± 1.79b	3.57 ± 1.16c	2.31 ± 0.54c
SO_4_^2−^ (g/kg)	13.44 ± 3.03a	12.09 ± 3.50a	7.56 ± 1.33b	5.02 ± 2.38b	4.81 ± 2.29b
Ca^2+^ (g/kg)	2.19 ± 1.04a	2.04 ± 1.05a	0.63 ± 0.35b	1.62 ± 0.86ab	1.72 ± 0.44ab
Mg^2+^ (g/kg)	0.37 ± 0.10a	0.47 ± 0.09a	0.20 ± 0.04a	0.19 ± 0.13a	0.16 ± 0.09a
Na^+^ (g/kg)	4.06 ± 2.24a	4.04 ± 2.24a	1.53 ± 1.00b	2.44 ± 0.90b	1.29 ± 1.01b
K^+^ (g/kg)	0.41 ± 0.34a	0.33 ± 0.27a	0.14 ± 0.12a	0.15 ± 0.08a	0.13 ± 0.10a

Letters above means represent the results of pairwise contrasts betweenthefive community classes.

All data are mean±SD. DistR is distance from the river channel to the quadrat; GWD is the groundwater depth; GS is the groundwater salinity; SM is the soil moisture; TS is the soil total salt; EC is the soil electrical conductivity; GEC is the groundwater electrical conductivity, the conversion factor is 0.515.

### DCCA analysis of the plant community and environmental factors

**Figure 4:**
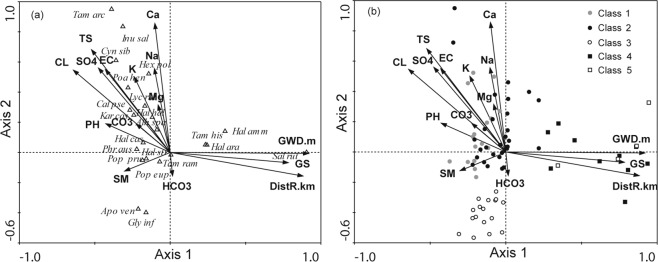
DCCA analysis of data from 22 plant species in upper reaches of Tarim River. Species names are listed in Table 1. (a) species are shown as triangles and labeled with their first three letters of the generic name and first three letters of the specific name, and environment characteristics are shown as arrow (where the DistR is the distance from the river channel to the quadrat, GWD is the groundwater depth, GS is the groundwater salinity, SM is the soil moisture, TS is the soil total salt; EC is the soil electrical conductivity); (b) DCCA analysis of plant quadrats of different classes and environment characteristics. The quadrats are divided into five classes (same as Table 2).

The results of the DCCA are displayed in ordination diagrams, with 77 quadrats or 22 species (Fig. 4). The triangles represent the species, and the vectors represent the 15 environmental parameters. The eigenvalues of the first two ordinations were 0.935 and 0.832. The first DCCA represents a gradient with increasing groundwater depth, distance from the river channel to the quadrat, and groundwater salinity, while soil moisture declines from left to right. The corresponding plant communities shift from classes 1 and 2 to classes 5 and 4. This suggests that plant community changes from high water consumers to drought-tolerant species. The community structure shifts from a tree-shrub-herb structure to a tree-herb or shrub-herb structure. The dominant plant species changed from *P. euphratica*, *T. ramosissima* and *L. ruthenicum* to *P. euphratica* or *T. hispida* as the distance from the river channel increased (Fig. 4a; A1). The composition of the herbaceous species changed from *P. australis*, *K. caspia*, *H. strobilaceum* and *C. pseudophragmites* to *S. ruthenica* and *H. arachnoideus*.

The second DCCA represents a gradient within declining soil salt (pH, EC, TS, CO_3_^2−^, Cl^−^, SO_4_^2−^, Ca^2+^, Mg^2+^, Na^+^, and K^+^), while groundwater salinity increases from top to bottom. The plant communities changed from classes 2 and 1 to class 3. The community structure changed from a tree-shrub-herb structure to a tree-shrub structure. The dominant species of the plant communities showed almost no change, but the soil salinity and groundwater salinity affected the herbaceous layer. There were few herbaceous plants, such as *A. venetum* and *G. inflata*, in class 3.

### PCA of the environmental factors in the different plant communities

Groundwater depth, groundwater salinity, soil moisture and soil salinity in the five plant communities were assessed using PCA (Tables 4 and 5). Five principal components (*g*_1_*, g*_2_*, g*_3_*, g*_4_, and *g*_5_) were extracted with eigenvalues > 1.0, and their cumulative contribution rate reached 95.95%. The orders of the comprehensive appraisal value scores of the environmental factors were as follows: class 1 > class 2 > class 3 > class 4 > class 5 (Table 5), which is consistent with the plant diversity index result (Fig. 3).

**Table 4:** Total variance explained and component matrixes (five principal component selected) for 15 parameters from the five plant communities using PCA.

Component	Initial eigenvalues			Extraction sums of squared loadings		
	Eigenvalues (λ)	Variance (%)	Cumulative variance (%)	Eigenvalues (λ)	Variance (%)	Cumulative variance (%)
1 DistR. km	7.28	46.86	46.86	7.28	46.86	46.86
2GWD. m	4.19	15.21	62.07	4.19	15.21	62.07
3GS (g/L)	2.28	13.44	75.51	2.28	13.44	75.51
4 SM (%)	1.52	10.22	85.73	1.52	10.22	85.73
5 TS (g/kg)	1.19	8.60	95.95	1.19	8.60	95.95
6 PH	0.34	2.66	98.61			
7 EC (ms/cm)	0.18	1.39	100.00			
8 CO_3_^2−^ (g/kg)	4.75E-16	3.65E-15	100.00			
9 HCO_3_^−^ (g/kg)	1.51E-16	1.16E-15	100.00			
10 Cl^−^ (g/kg)	1.34E-16	1.03E-15	100.00			
11 SO_4_^2−^ (g/kg)	− 5.20E-17	− 4.00E-16	100.00			
12 Ca^2+^ (g/kg)	− 1.45E-16	− 1.12E-15	100.00			
13 Mg^2+^ (g/kg)	− 1.92E-16	− 1.48E-15	100.00			
14 Na^+^ (g/kg)	− 2.99E-16	− 2.30E-15	100.00			
15 K^+^ (g/kg)	− 4.00E-16	- 3.08E-15	100.00

**Table 5:** Principal component score matrix of 15 parameters from five plant communities and their comprehensive appraisal value (*g*) of groundwater and soil salinity.

Class	Principal component scores					Comprehensive appraisal value (*g*)	Rank
	*g*_1_	*g*_2_	*g*_3_	*g*_4_	*g*_5_		
Class 1	17.04	0.82	3.20	7.61	1.89	9.03	1
Class 2	14.01	−1.04	3.38	−3.30	2.01	6.24	2
Class 3	9.27	−2.86	−1.80	−4.37	1.32	2.81	3
Class 4	−1.52	7.34	8.36	1.05	0.96	2.52	4
Class 5	−1.27	3.74	5.85	0.97	0.88	1.35	5

## Discussion

The Tarim River is China’s largest river and is the world’s fifth largest endorheic river^20^. In the present study, 22 plant species were found in the upper reaches of the Tarim River, which is higher than the number of species recorded in the lower reaches^33^. The plant species richness in the Tarim River is similar to that in the Syr Darya and Amu Darya Rivers^34,35^ but is low compared to that in the Gurbantünggüt Desert of the Junggar Basin in China^36^. In this study, the plant importance value analysis showed that *P. euphratica* and *T. ramosissima* were the most significant species in the tree and shrub layers, respectively (Table 1). This suggests that *P. euphratica* and *T. ramosissima* are dominant species in the upper reaches of the Tarim River, which corroborates the study of Hao *et al*.^33^ in the lower reaches of the Tarim River. It is possible that *P. euphratica* and *T. ramosissima* utilize a “sit-and-wait” strategy to avoid the disturbances from river runoff, resulting in them being the dominant species in the riparian plant communities of the upper and lower reaches^37^.

In the riparian forests of the upper reaches of the Tarim River, 2 trees, 7 shrubs and 13 herbaceous species were found during the survey. TWINSPAN successfully distinguished the riparian forests into five classes, which is greater than that recorded in the lower reaches of the Tarim River^38^. A partial overlap in species composition among the five classes was identified, indicating that some species exhibit broad environmental tolerance. For example, the keystone species *P. euphratica* and *Tamarix* spp. can exist from the riverside to the oasis and desert margins^9,12^. It is possible that *P. euphratica* and *Tamarix* spp. are flood-tolerant species^37,39,40^ and that they have evolved a unique allocation strategy that allows them to withstand flooding. For example, they often lose part of their aboveground biomass during flooding and increase the allocation of biomass to their roots during favorable times^41^. This supports the storage effect theory that carbohydrates stored in belowground tissue during favorable times allow the plants to survive flooding. Additionally, *P. euphratica* and *Tamarix* spp. are drought-tolerant species, and *P. euphratica* was found to growing in locations with a groundwater depth of up to 13 m (Table 3), which was in agreement with the finding of Gries *et al*.^42^ and Thomas *et al*.^43^. *Tamarix* spp. were found growing in locations with a groundwater depth of more than 14 m and had a greater ability than that of the other species to extract water from a relatively dry soil^5^, which was in agreement with the results presented by Gries *et al*.^42^.

Water availability plays an important role in the composition and distribution of plant communities, particularly in arid and semi-arid regions^43^. The DCCA indicated that the plant communities changed from classes 1and 2 to classes 5 and 4, transitioning from a tree-shrub dominated communities to a tree or shrub dominated communities as the water conditions changed from good to poor. The herbaceous species changed from *P. australis*, *K. caspia*, *H. strobilaceum* and *C. pseudophragmites* to *S. ruthenica* and *H. arachnoideus* as the distance from the river channel increased. This may be because herbaceous plants with shallow root systems are eliminated when the groundwater depth is too deep^45,46^. However, the herbaceous species *S. ruthenica* and *H. arachnoideus* can grow in desert habitats. These two herbaceous species exist under the dominant species *P. euphratica* and *Tamarix* spp., which have a significant “fertility island” effect^44^. For example, the plants trap nutrient rich sediments transported during floods, provide a sheltered microhabitat and reduce the surface temperature of the soil in the summer^47^. Therefore, *P. euphratica* and *Tamarix* spp.were the “nurse plants” for these two herbs.

In this study, the dominant species showed almost no change when the plant communities changed from classes 1 and 2 to class 3 as the soil salinity changed from high to low. This may be because the dominant species, *P. euphratica* and *T. ramosissima*, have deep roots and are able to access the less saline, shallow groundwater. However, soil salinity affected the herbaceous layer. This may be because the herbaceous plants may be more affected by changes in surface soil salinity because their roots are unable to access the less saline groundwater. There were few herbaceous plants, such as *A. venetum* and *G. inflata*, in class 3. *A. venetum* not only grows in class 3 but is also found in classes 1 and 2. This result indicated that *A. venetum* is distributed widely across the study area. Therefore, the different soil salinity requirements (i.e., niche differences) of the herbaceous plant reflect the soil salinity can determine the distributions of the herbaceous plants.

Environmental variability is considered to have an important influence on species diversity due to its effects on plant growth, development, and regeneration^37,45,48^. In this study, we analyzed the environmental characteristics of different plant communities using principal component analysis (PCA). The comprehensive appraisal value scores of the environmental factors of the five communities were ranked as follows: class 1 > class 2 > class 3 > class 4 > class 5. Plant diversity may change in response to environmental gradients^49^. The quadrats in classes 1 and 2 were mainly distributed close to the river; this area is associated with shallow groundwater depth, low groundwater salinity, and high soil moisture and soil salinity. These environmental factors have positive effects on species diversity^14,20,21,49^. It is also possible that the quadrats close to the river experienced flooding disturbances, and as plant diversity is highest at moderate flooding stress, this supports the intermediate disturbance hypothesis^2,50^. The quadrats in classes 4 and 5 were mainly distributed at the edge of the desert; this area is characterized by a deep groundwater depth, high groundwater salinity, and low soil moisture. These environmental factors have negative effects on species diversity^10,38,43^. The seedlings of the dominant species, *P. euphratica* and *Tamarix* spp., were mainly established in a moist environment near the river channel^5^, while almost no seedlings had established at the edge of the desert^12^. The spatial variation in key environmental variables resulted in different plant assemblages at the patch scale which contributes to plant diversity at larger spatial scales. Therefore, the environmental factors that are creating the habitat heterogeneity which in turn affects plant diversity.

The extent of riparian vegetation has declined significantly in response to changes in the environment. The area of the tugai forest declined by 3.0 × 10^5^ ha from 1958 to 1978 in the Tarim Basin and by 4.3× 10^5^ ha from 1950 to 1998 in the Aral Sea Basin^51^. Furthermore, the *P. euphratica* forest has decreased from 5.4 × 10^4^ hm^2^ to 0.67 × 10^4^ hm^224^, and this species has been listed as an endangered national level three protected plant in China^48^. The tugai forests thus constitute a highly threatened ecosystem^52^. Plant species diversity and richness are considered to be the primary objectives of successful restoration^53^. Our study demonstrates that the plant diversity indices in classes 1 and 2 were higher than those in classes 4 and 5 (Fig. 3). This might indicate that the environmental factors of classes 1 and 2, such as groundwater depth, groundwater salinity, and soil moisture, were more suitable for plant growth than those of classes 4 and 5. Classes 1 and 2 were characterized as tree-shrub-herb structures, which are highly stable and have a stronger sand stabilization ability than that of classes 4 and 5^38^. However, classes 4 and 5 were characterized as shrub-herb and tree-herb structures, respectively. These structures are also effective at sand stabilization. Therefore, we suggest that to protect the riparian plant community, all habitats, rather than some, should be considered for conservation. Conservation managers need to ensure that a sufficient amount of habitat is maintained for the structural and functional sustainability of the riparian forest. This finding has great significance for the restoration and protection of damaged desert riparian ecosystems.

## Material and methods

### Study area

In this study, the upper reaches of the Tarim River were selected as the study area (Fig. 1). The elevation ranges from 900 m to 1050 m above sea level; the annual precipitation ranges from 50 mm to 70 mm; and the annual pan evaporation is more than 2100 mm^12^. The average annual temperature is 10.6–11.5 °C, with a minimum and maximum temperature of −27.5 °C and 43.6 °C^25^, respectively. The vegetation mainly includes *Populus euphratica*, *Tamarix* spp., and *Alhagi sparsifolia*^5,12^.

### Plant quadrats and measurements

In this study, the survey work was performed in July 2016 in the upper reaches of the Tarim River. There are obvious differences in plant diversity from the river channel to the edge of the desert in this area^12^. The distance from the river channel to the edge of the desert is approximately 30 km^12^. Therefore, to fully understand the correlations between the plant assemblage and the environmental variables, 77 quadrats were investigated. Nuclear magnetic resonance (GMR, Vista Clara Inc., WA, USA) and ground penetrating radar (RIS-2K, IDS Ingegneria dei Sistemi S.p.A., Italy) were used to ascertain the groundwater depth. Groundwater salinity (GS) was determined based on the method reported in Zhou^26^. The size of the plant quadrats was 100 m × 100 m. Sixteen sub-quadrats of 25 m × 25 m were used for recording the characteristics of the tree and shrub plants in each plant quadrat. For example, the diameter of trees at breast height (DBH) (breast height = 1.3 m) was recorded for each tree (≥5 cm DBH)^12^. The height, width, and number of species were recorded for the tree layer and shrub layer. Four sampling quadrats of 5 m×5 m were used for recording the number, height, and width of herbs in each sub-quadrat (25 m ×25 m). A GPS was used to record the quadrat locations.

### Soil sampling and measurement

In each quadrat, the soil samples were randomly collected from five location in the upper 20 cm soil layer. The samples were air-dried and then passed through 2 mm sieves before the soil analyses. The soil pH, electrical conductivity (EC), and total salt (TS) were determined using a suspension of the soil sample and deionized water (ratio of 1: 5)^27,28^. A glass electrode pH meter was used to determine the soil pH^27^, the dry residue method was used to determine the TS, anda conductivity meter was used to determine the EC^28^. The neutral double indicator method was used to test for bicarbonate (HCO_3_^−^) and carbonate (CO_3_^2−^). AgNO_3_ titration and EDTA indirect titration were used to determine sulfate (SO_4_^2−^) and chloride (Cl^−^), respectively. Complexometry was used to determine the calcium (Ca^2+^) and magnesium (Mg^2+^), the flame photometer method was used to determine the sodium (Na^+^) and potassium (K^+^), and the soil moisture was determined by oven-drying the samples.

### Calculation of diversity

The plant species diversity was determined using the Simpson diversity index (*D*_*S*_)^29^, Shannon-Weiner diversity index (*H*)^30^, and Pielou evenness index (*J*_*SW*_)^31^. The following formulae were used:

1 $${D}_{S}=1-{\sum {P}_{i}}^{2}$$

2 $$H=-\,\sum {P}_{i}\,\mathrm{ln}\,{P}_{i}$$

3 $${J}_{SW}=H/lnS$$

where *S* is the number of species, and *N* is the number of individuals of all the species in a community. In ${P}_{i}={n}_{i}/N$, *n*_*i*_ is the importance value of species *i* in a community, and *N* is the sum of the importance values of all the species.

### Calculation of the relationship between the environment and plant community

Two-Way indicator species analysis (TWINSPAN) method was used to identify the riparian plant communities based on the importance value of the species in all the quadrats^7^. The plant importance value was calculated according to the following equation^7,12^:

4 $${\rm{Importance}}\,{\rm{value}}=({\rm{relative}}\,{\rm{density}}+{\rm{relativefrequency}}+{\rm{relative}}\,{\rm{dominance}})/3$$

The diameter at breast height was used for the determination of the relative dominance of the trees, while basal coverage was used for the shrubs and herbs.

TWINSPAN was performed using PC-ORD5.0. Detrended canonical correspondence analysis (DCCA) wasused to analyze the relationship between the environmental factors and the plant community composition^7^. Two data matrices are required for DCCA. Oneisa species-by-quadrats matrix, and the other one is an environment-by-quadrats matrix. The ordination program CANOCO 4.5 was used to perform the DCCA^7^. The differences in the species diversity indices, groundwater and soil salinity between the five plant community classes analyzed here were compared individually using multiple comparisons [Tukey’shonest significant difference (HSD) tests at *P* < 0.05].

Principal component analysis (PCA)^32^ method was used to assess the comprehensive appraisal value (*g*) of groundwater and soil salinity in different plant communities. The following formulae was used:

5 $$g=\mathop{\sum }\limits_{i=1}^{n}({x}_{i}/\mathop{\sum }\limits_{i=1}^{n}{x}_{i}){g}_{i}$$

where *g* is the value of the comprehensive appraisal of the environmental characteristic, *n* is the number of principal components, *x*_*i*_ is the eigenvalue of the *i*th principal component, ${x}_{i}/\mathop{\sum }\limits_{i=1}^{n}{x}_{i}$is the weighing factor of the *i*th principal component, and *g*_*i*_ is the *i*th principal component score. All the principal components extracted from the variables with eigenvalues > 1.0 and a cumulative contribution rate of extraction ≥ 85% were retained^32^.

## Supplementary information

Supplementary information
