# Supplementary material for: Impact of groundwater depth and soil salinity on riparian plant diversity and distribution in an arid area of China
Source: Sci Rep. 2020 Apr 29;10:7272. doi: 10.1038/s41598-020-64045-w (PMC7190620; doi:10.1038/s41598-020-64045-w)

Impact of groundwater depth and soil salinity on riparian plant diversity and distribution in an arid area of China

Yong Zeng[1,2,3], **Chengyi Zhao**[4,1*], **Fengzhi Shi** [1], **Michael Schneider**[5], **Guanghui Lv**[2], **Yan Li**[1]

[1]*State Key Laboratory of Desert and Oasis Ecology, Xinjiang Institute of Ecology and Geography, Chinese Academy of Sciences, Urumqi 830011, Xinjiang, China.* [2]*College of Resources and Environmental Sciences, Xinjiang University, Urumqi 830046, Xinjiang, China.* [3]*University of Chinese Academy of Sciences, Beijing 100049, China.* [4]*Nanjing University of Information Science and Technology, Nanjing 210044, China.* [5]*Earth Sciences, Freie Universität Berlin (FUB), Malteserstr. 74-100, 12249 Berlin, Germany*

[*] *For correspondence author. E-mail* zhaocy@nuist.edu.cn

Telephone number +86 13579286610

Figure A1 Five plant communities in upper reaches of Tarim River. *Populus euphratica*, *Tamarix ramosissima* and *Lycium ruthenicum* were the dominant species in Class 1; *P. euphratica* and *T. ramosissima* were the dominant species in Class 2; *P. euphratica* and *T. ramosissima* were the dominant species in Class 3; *T. hispida* is the dominant species in Class 4; *P. euphratica* is he dominant species Class 5. Photo Y. Zeng and F. Shi in July 2016.

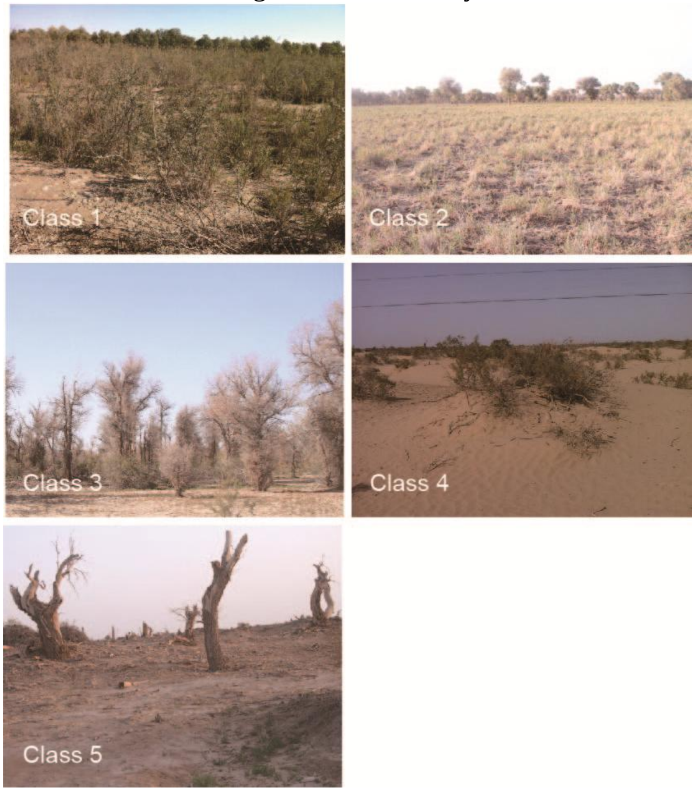

Supplement: Supplementary file 1 — Supplementary information [file 41598_2020_64045_MOESM1_ESM.pdf]
